# Contribution of the Lateral Prefrontal Cortex to Cognitive-Postural Multitasking

**DOI:** 10.3389/fpsyg.2018.01075

**Published:** 2018-07-06

**Authors:** Christine Stelzel, Hannah Bohle, Gesche Schauenburg, Henrik Walter, Urs Granacher, Michael A. Rapp, Stephan Heinzel

**Affiliations:** ^1^Division of Social and Preventive Medicine, University of Potsdam, Potsdam, Germany; ^2^Experimental Psychology, International Psychoanalytic University Berlin, Berlin, Germany; ^3^Division of Training and Movement Science, University of Potsdam, Potsdam, Germany; ^4^Department of Psychiatry and Psychotherapy, Charité – Berlin Universitätsmedizin, Corporate Member of Free University of Berlin, Humboldt University of Berlin, Berlin Institute of Health, Berlin, Germany; ^5^Berlin Center for Advanced Neuroimaging, Charité – Berlin Universitätsmedizin, Berlin, Germany; ^6^Clinical Psychology and Psychotherapy, Free University of Berlin, Berlin, Germany

**Keywords:** balance, dual task, fMRI, postural control, working memory

## Abstract

There is evidence for cortical contribution to the regulation of human postural control. Interference from concurrently performed cognitive tasks supports this notion, and the lateral prefrontal cortex (lPFC) has been suggested to play a prominent role in the processing of purely cognitive as well as cognitive-postural dual tasks. The degree of cognitive-motor interference varies greatly between individuals, but it is unresolved whether individual differences in the recruitment of specific lPFC regions during cognitive dual tasking are associated with individual differences in cognitive-motor interference. Here, we investigated inter-individual variability in a cognitive-postural multitasking situation in healthy young adults (*n* = 29) in order to relate these to inter-individual variability in lPFC recruitment during cognitive multitasking. For this purpose, a one-back working memory task was performed either as single task or as dual task in order to vary cognitive load. Participants performed these cognitive single and dual tasks either during upright stance on a balance pad that was placed on top of a force plate or during fMRI measurement with little to no postural demands. We hypothesized dual one-back task performance to be associated with lPFC recruitment when compared to single one-back task performance. In addition, we expected individual variability in lPFC recruitment to be associated with postural performance costs during concurrent dual one-back performance. As expected, behavioral performance costs in postural sway during dual-one back performance largely varied between individuals and so did lPFC recruitment during dual one-back performance. Most importantly, individuals who recruited the right mid-lPFC to a larger degree during dual one-back performance also showed greater postural sway as measured by larger performance costs in total center of pressure displacements. This effect was selective to the high-load dual one-back task and suggests a crucial role of the right lPFC in allocating resources during cognitive-motor interference. Our study provides further insight into the mechanisms underlying cognitive-motor multitasking and its impairments.

## Introduction

Multitasking comprises a temporal overlap in the performance of different tasks (Wickens, 1980; Pashler, 1994) and performance costs in multitasking are often assumed to depend on the recruitment of common resources in both tasks (Kahneman, 1973; Tombu and Jolicoeur, 2003). While concurrent performance of two cognitive tasks has been associated with additional processing demands in the lateral prefrontal cortex (lPFC; D’Esposito et al., 1995; Schubert and Szameitat, 2003), little is known about the role of the lPFC in the concurrent processing of cognitive tasks and complex motor tasks such as keeping balance on an unstable surface.

Human postural control affords the control of the body’s position in space for the purpose of stability and orientation (Shumway-Cook and Woollacott, 2017). Adequate postural alignment is not just a passive state but requires targeted muscle activation through the interplay of the peripheral and central nervous system, including information processing in the proprioceptive, cutaneous, visual, and vestibular systems (Peterka, 2002; Baudry, 2016). Evidence from several methodological approaches indicates an involvement of higher (central) level processes in postural control (Jahn et al., 2004; Jacobs and Horak, 2007; Taube et al., 2008; Lau et al., 2014; Papegaaij et al., 2014; Varghese et al., 2015; Wittenberg et al., 2017). Besides sensory and motor systems, there is evidence that the lPFC is involved in human postural control as well. For example, functional imaging revealed an activation of the lPFC in imagined stance (Jahn et al., 2004). Moreover, balance training in young adults resulted in increases in prefrontal gray matter volume and prefrontal fiber connections (Taubert et al., 2010). Also, using functional near-infrared spectroscopy (fNIRS), Mihara et al. (2008, 2012) provided direct evidence for prefrontal contributions to human postural control. This prefrontal contribution may in turn be responsible for interference with cognitive control tasks known to recruit regions of the lPFC as well (Duncan and Owen, 2000; Fuster, 2001).

A number of behavioral studies showed interference effects during the concurrent performance of motor tasks involving postural control or gait and cognitive control tasks, with particularly pronounced effects in old adults (Woollacott and Shumway-Cook, 2002; Rapp et al., 2006; Granacher et al., 2011; Boisgontier et al., 2013). Evidence for an association of lPFC activation and these interference effects is limited and mostly restricted to regionally unspecific methodological approaches (Holtzer et al., 2011; Doi et al., 2013; Zhou et al., 2014; Little and Woollacott, 2015). For example, an fNIRS study revealed that dual-task “walking while talking” is associated with higher prefrontal activation than single-task walking in both young and old adults (Holtzer et al., 2011). In this study, the dual-task condition included to walk at self-selected gait speed while concurrently naming every second letter of the alphabet. During dual-task compared to single-task walking, higher oxygenation levels in the lPFC were present in young and old individuals. Another fNIRS study tested the role of individual differences in working memory capacity during the concurrent performance of a postural task and a Stroop task (Fujita et al., 2016). Findings from this study showed dual-task-related lPFC recruitment to be associated with working memory capacity with greater dual-task effects in high span participants. Furthermore, transcranial direct current stimulation (TDCS) over the lPFC improved balance and gait performance in cognitive-motor dual-task situations involving a serial subtraction task in young adults (Zhou et al., 2014).

While these studies show a general involvement of the lPFC in cognitive-motor dual tasks, little is known about the specific lPFC sub-regions that are involved in the processing of cognitive-motor dual tasks. This could be investigated by using spatially more precise neuroscientific methods such as functional magnetic resonance imaging (fMRI) and by applying a task design that allows investigating specific cognitive demands related to multitasking. Clearly, a methodological limitation of using fMRI in balance research is that it is not possible to measure brain activity and whole body balance performance within the same session [but see Al-Yahya et al. (2016), Papegaaij et al. (2017) for recent approaches using balance and gait simulation tasks]. However, by correlating individual variability in dual-task-specific brain activity (i.e., cognitive dual-task compared to single-task activity) with individual variability in performance costs in a cognitive-postural triple vs. dual task, the cognitive and neural underpinnings of cognitive-postural interference can be deduced.

In a cross-sectional study, as part of a larger-scale multimodal study, we tested potential associations between an lPFC demanding dual one-back working memory task using fMRI and performance costs in a cognitive-postural task measured on a force plate in healthy young adults. Of note, fMRI and force plate testing was realized in different test sessions. Several studies showed the degree of right lPFC recruitment to be associated with individual differences in various cognitive control tasks (Locke and Braver, 2008; Jimura et al., 2010; Heinzel et al., 2016). In these studies, either intra- or inter-individual increases in right lPFC activity were related to better task performance. This has been associated with increased neural effort in cognitive tasks by which individuals improved performance despite increases in cognitive demands or lower working memory capacity (Eysenck et al., 2007; Barulli and Stern, 2013). Accordingly, we expected dual-task-specific activity in the right lPFC to be associated with cognitive-postural performance costs. More specifically, we expected increased right lPFC activity in dual compared to single one-back tasks to be associated with relative performance costs in a postural task that is performed concurrently with a dual-one back task, that is when cognitive task load is high.

## Materials and Methods

### Participants

Thirty-one young adults participated in this study after verbal instructions were provided and written informed consent was given. Participants were mainly recruited through student mailing lists at the University of Potsdam, Germany, in the context of a large-scale study also involving electroencephalographic (EEG) measurement. Two participants had to be excluded from the analysis – one due to technical failure of the force plate and one due to strong movement during MRI measurement. Thus, the final sample consisted of 14 male and 15 female participants with a mean age of 24.8 years (range: 19–30 years).

All participants were healthy with no adverse signs or self-report of neurological or psychiatric disorders, no hearing impairments, normal or corrected-to-normal vision. Furthermore, suitability for MRI measurement was assessed through self-report.

Participants came to the biomechanics laboratory of the Division of Training and Movement Sciences, University of Potsdam for two test occasions and thereafter to the Berlin Center for Advanced Neuroimaging, Charité Berlin for MRI measurement. Test sessions were separated by a minimum of 1 week and a maximum of 4 weeks. Before the first test session, participants were screened for eligibility via telephone interviews and received a set of questionnaires via mail.

This study was designed according to the latest version of the Declaration of Helsinki and was approved by the local ethics committees of the University of Potsdam and the Charité Universitaetsmedizin Berlin, Germany. Study participation was reimbursed monetarily with 60 € for three test sessions.

### Experimental Tasks

**FIGURE 1:**
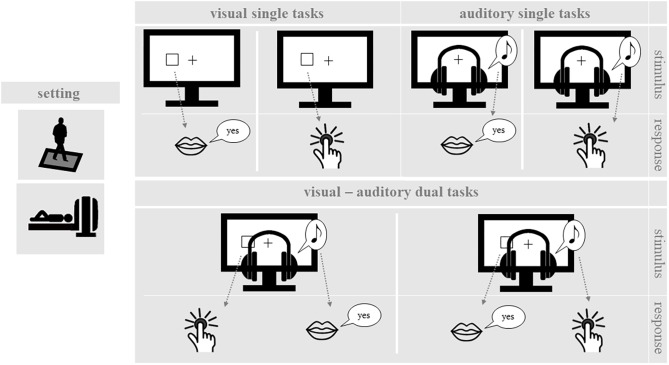
One-back working memory tasks. In the single one-back tasks, participants decided for visual or auditory stimuli whether they are identical to the stimulus in the trial before. In the dual one-back tasks visual and auditory stimuli were presented simultaneously and participants made the one-back decision for both stimuli.

While standing on the force plate and during MRI measurement, participants performed single one-back tasks and dual one-back tasks, which covered a range of input stimuli and output responses (see **Figure 1**). For each delivered stimulus, participants had to decide whether it was the same as the previous one (one-back). Throughout testing, participants wore headphones with an attached microphone. During the balancing task on the force plate, all participants were equipped with a response key in their right hand, which allowed them to press a button with their right thumb. Inside the scanner, participants used their right index finger. The following cognitive and postural tasks were applied:

#### Single One-Back Tasks

Participants performed different versions of a spatial one-back working memory task. An instruction trial before each block indicated them the task for the following block. Input stimuli were either visual or auditory and responses were given either manually or vocally. The stimulus duration was 500 ms followed by a fixation inter-stimulus interval of 1500 ms. Task blocks consisted of 16 trials, including 5 one-back targets and 11 non-targets in pseudo-random order. By combining the different input stimuli and output responses, there were four different types of cognitive single one-back tasks.

##### Visual-manual one-back task

The target display consisted of a black background with a white fixation cross in the center. Visual stimuli were presented as white squares located at six different spots on the screen (up, center, down), three on each side of the fixation cross. Participants were instructed to respond fast and correctly by pressing a button whenever the position of the current square was the same as in the preceding trial.

##### Auditory-vocal one-back task

Three different tones were presented at frequencies of 200, 450, 900 Hz via headphones while a static fixation cross was displayed on the screen. The tones were presented either to the left or the right ear, resulting in six different stimuli. As in the visual task, participants were instructed to respond fast and correctly, when the same tone was presented to the same ear in trials *n* and *n*-1. Participants were instructed to respond vocally to target stimuli by saying “yes” (German: “Ja”).

##### Visual-vocal one-back task

The target display and stimulus presentation were the same as in the visual-manual one-back task. However, in this case participants had to respond vocally to target stimuli by saying “yes” (German: “Ja”).

##### Auditory-manual one-back task

Targets and stimulus presentation were the same as in the auditory-vocal condition. However, during this experimental condition participants had to respond manually to target stimuli via button press.

#### Dual One-Back Tasks

In dual-task blocks, participants performed two one-back tasks simultaneously. For this purpose, a visual and an auditory stimulus were presented simultaneously for 500 ms, followed by a 1500 ms inter-stimulus interval. Participants were instructed to decide for both presented stimulus modalities whether the stimulus was identical or not to the prior stimulus (dual one-back task). In dual one-back task blocks, both the visual-manual and the auditory-vocal task were performed simultaneously or the visual-vocal and the auditory-manual task were performed simultaneously. Accordingly, there was no overlap in stimulus modality or response modality in either dual one-back task. For each task block, five one-back targets were presented, i.e., two or three in the visual modality and two or three in the auditory modality. One-back targets were presented either in the auditory or in the visual modality but never simultaneously.

#### Postural Baseline Task on Force Plate

With their arms hanging loose to the sides of the body, participants were instructed to stand as still as possible in semi-tandem stance on an unstable surface (i.e., balance pad) with the dominant leg posterior to the non-dominant leg. To determine participants’ dominant leg, we asked them to softly kick a ball placed approximately 1.5 m right in front of the participant. We registered the kicking leg as the dominant leg. Further, participants answered two questions of the lateral preference inventory (Coren, 1993) concerning leg dominance: (i) which leg would you use to pick something up from the ground? and (ii) which leg would you use to step on a burning cigarette on the ground? We defined the dominant leg as the leg, which was the one that was most often mentioned/used in these three situations. The balance pad was placed on a one-dimensional force plate (Leonardo 105 Mechanograph^®^; Novotec Medical GmbH Pforzheim, Germany) in order to measure total CoP displacements during testing. Participants had to keep their head straight and their gaze fixated either on a stable visual stimulus (*stable fixation condition*) or on a dynamic visual stimulus (*dynamic fixation condition*). In the stable fixation condition, participants had to focus their gaze on a fixation cross which was presented in the center of the screen. In the dynamic fixation condition, a fixation cross and an ampersand symbol (“&,” fontsize: 54) were displayed alternately in the center of the screen, with presentation times matched to presentation times in the cognitive tasks (i.e., 500 ms ampersand, 1500 ms fixation). Here, we only report the dynamic fixation condition, as our pilot studies revealed higher postural instability during stable fixation.

### Procedure

The first test day comprised a neuropsychological screening procedure, including tests for vision and hearing abilities and several specific neuropsychological and motor tests (e.g., Digit Span, Trail Making A & B, Timed Up & Go Test). These neuropsychological tests were included to compare the young adults as a control group to a cohort of old adults who underwent further experimental sessions as well as a cognitive-motor training procedure (to be reported elsewhere). At the end of the session, participants practiced the single and dual one-back tasks, with two blocks including 32 trials for each single one-back task and four blocks of 32 trials for each dual one-back task after detailed instructions.

On the second test day, participants performed the experimental tasks as outlined above which included the assessment of total CoP displacements while standing on the force plate and the concurrent recording of EEG data using a mobile 64-channel EEG system (EEG data not reported here). The experiment consisted of two sessions with six runs each. Within each run, three one-back task blocks were performed (two single one-back tasks, one dual one-back task). In each session, three runs were conducted in standing upright position and three while sitting upright, performed in an alternating mode. The sessions differed in the specific stimulus-response mappings to be performed, i.e., in one session only visual-manual and auditory-vocal tasks were realized, in the other session only visual-vocal and auditory manual tasks (see Stelzel et al. (2017) for more details).

All participants performed both sessions in direct succession with a short break in-between and the order of the sessions was counterbalanced between participants. All participants started in the semi-tandem stance condition. The standing condition always began with one stable fixation block, followed by a dynamic fixation block (33 s each to match the duration of the cognitive tasks). Thereafter, the three cognitive task blocks followed (two single one-back blocks and one dual one-back block, the order counterbalanced across runs, 33 s each) which were again followed by one dynamic fixation block and one static fixation block. Each cognitive task block included 16 trials. While sitting, only the three cognitive task blocks were performed in the same order as in the preceding standing condition.

Participants practiced the relevant tasks one more time at the beginning of the second test day right before the experimental session in sitting position (one task block per single one-back task, two task blocks per dual one-back task) started.

The third test day included the MRI measurement. Here, the same single and dual one-back tasks were performed in a block design. There were six runs for this experiment with six task blocks per run. As in the previous session, visual-manual, and auditory-vocal tasks were performed in three runs and visual-vocal and auditory manual tasks in the other three runs. The different types of runs were alternated. Each run consisted of four single-task blocks and two dual-task blocks. Single- and dual-one-back task blocks were again counterbalanced in their order across runs. The order of runs was counterbalanced across participants. Block duration was 34 s, inter block intervals were 12 s (gray fixation cross), followed by 2 s of instructions for the next task block.

During the MRI session, participants performed three additional tasks: the MRI session always started with a zero-back task. After the one-back task, which is subject of the current paper, a resting state measurement and a task switching experiment were conducted. These data will be reported elsewhere.

### Performance Assessment and Analysis

#### Cognitive Performance

Visual and auditory stimuli were presented, and manual and vocal responses were recorded via Presentation software^1^. Performance data of the cognitive tasks were calculated as p(Hit)-p(False alarm). Vocal and manual responses were recorded during the experiment for the period of each one-back trial duration (2 s). Vocal data were analyzed offline with a self-developed Matlab tool (MathWorks; Natick, MA, United States). The custom-made tool (Reisner and Hinrichs, 2016) was developed to facilitate automated identification of trials with correct vocal responses and to extract reaction time (RT) latencies based on simple signal amplitude measurement. The tool was validated successfully via manual coding of vocal responses (Cohens Kappa = 0.941, *p* < 0.001). Due to technical failure during recording, the vocal data of eight young participants were not recorded properly in the force plate session and could not be analyzed. These participants were excluded from all analyses including one-back performance data from the force plate session but were included in the analysis of fMRI and CoP data. Cognitive performance data were averaged for all single tasks and dual tasks, respectively.

These data were then subjected to a general linear model (GLM), with two within subject factors with two levels each: 1. Force plate vs. MRI × 2 single one-back vs. dual one-back task. In addition to these performance measures, mean RTs for correct target responses are reported.

Additionally, relative performance costs in p(hit)-p(false alarm) in the dual one-back task were calculated in relation to the single one-back task [(Single-Dual)/Single)^∗^100] to then calculate the correlation of cognitive performance costs with postural performance costs.

#### Balance Performance

Postural sway was assessed during semi-tandem stance (barefoot or with socks) on an unstable surface (i.e., balance pad) with the dominant leg posterior to the non-dominant leg. The balance pad (Airex^®^) was placed on a one dimensional force plate. Total CoP displacements (mm) were computed using CoP displacements in medio-lateral and anterior-posterior directions by means of the Pythagorean theorem. Assessment duration (34 s) was chosen in order to optimize reliability of postural sway measurement (Le Clair and Riach, 1996) and in accordance with the cognitive task requirements.

For statistical analysis, we ran an exploratory data analysis using JMP^®^ software (JMP^®^ 8, SAS Institute GmbH, Germany) to exclude outlier blocks for each participant. Outlier blocks were identified by box plot analyses on the subject level and defined as blocks which were outside the whiskers, that is blocks that were outside the range of <1st quartile – 1.5^∗^interquartile-range or >3rd quartile + 1.5^∗^interquartile range. Altogether, 2.8% outlier blocks were identified and excluded from further analyses.

Performance data of total CoP displacements for the baseline postural task (P), plus cognitive single one back task (CP), plus cognitive dual one-back task (CCP) were calculated by averaging CoP displacements of the respective conditions. Relative multiple task costs for total CoP-displacements were calculated for each run and averaged per condition according to the formula of Doumas et al. (2008). Thus, relative dual-task costs of total CoP displacements during single one-back performance were calculated as ([CP-P]/P) ^∗^ 100, and during dual one-back performance as ([CCP-P]/P) ^∗^ 100. To examine assumed effects of task load, we used paired t-tests (CP vs. CCP). All statistical analyses were processed using IBM SPSS Statistics, Version 22.0. Effect sizes (partial eta squared [$\eta_{\text{p}}^{2}$], Cohen’s *d*) are reported for all analyses to characterize the effectiveness of the experimental factors.

### fMRI Acquisition

All images were acquired using a 3 Tesla Siemens TIM Trio MRI scanner with blood oxygen level-dependent (BOLD) contrast and a 12-channel head coil at the Berlin Centre for Advanced Neuroimaging (BCAN, Berlin, Germany). Head motion was limited using foam head padding for comfortable stabilization. Participants were provided with earplugs and headphones to dampen scanner noise and enable communication. Experimental stimuli were presented with Presentation software (Neurobehavioral Systems, see footnote 1) and projected onto a screen positioned at the head end of the bore, viewable through a mirror attached to the head coil. Behavioral performance was also recorded with Presentation software via a fiber optic response keypad and an MRI-compatible microphone (FOMRI III^TM^+ microphone by Optoacoustics).

T2-weighted echo-planar images (EPI) were performed in six runs (echo time TE = 30 ms, flip angle = 78°, field of view = 24 cm, matrix size = 64 × 64, TR = 2 s, slice thickness = 3 mm, inter-slice gap = 0.75 mm). Each run contained 150 volumes with 33 axial slices each. All slices were oriented to the anterior–posterior commissure plane based on an auto-align procedure. Furthermore, field maps were acquired between the third and the fourth run of the experiment using the same slice prescriptions as for functional scans. After the experiment, a structural T1-weighted 3-D MPRAGE scan was performed (matrix size 256 × 256 × 192, slice thickness: 1.0 mm). Anatomical images were used for the normalization of the functional data to the Montreal Neurological Institute (MNI) atlas space.

### fMRI Data Analyses

All analyses of functional MRI data were performed with Statistical Parametric Mapping software (SPM12^2^). The functional volumes of each participant were first realigned and unwarped, then co-registered to the anatomical image. Participants with high movement (>1 mm within run, rotations > 1°) were excluded from further analyses. This was the case for only one participant. Subsequent preprocessing stages included segmentation of the anatomical images and spatial normalization of the functional datasets into standard MNI space by applying the parameters of the normalization of the anatomical image. Finally, functional data were smoothed with an 8 mm FWHM Gaussian kernel and high-pass filtered to a cut-off of 1/124 Hz during statistical analyses. An analytic design matrix was constructed modeling onsets and duration of each task condition for each participant and a GLM for serially auto-correlated data was applied (Friston et al., 1995). The functional volumes acquired during the six runs were treated as separate time series.

The data were analyzed as a block design including one covariate for each type of single one-back task (visual-manual, auditory-vocal, visual-vocal, auditory manual) and one for each dual one-back task (visual-manual and auditory-vocal, visual-vocal, and auditory-manual*)*, represented by boxcar functions with a duration of 34 s. Additional covariates were included for instructions (2 s), fixation periods (12 s) between the blocks and six movement parameters as covariates of no interest. Regression parameters were estimated using the classical restricted maximum likelihood (ReML) algorithm.

On the second level, one-sample *t*-tests were used to test for dual-task-specific activity, i.e., the contrast of all dual tasks with all single tasks. A cluster-wise family-wise error (FWE) correction was used to correct for multiple comparisons, with a threshold of *p* < 0.05 FWE at the cluster level (and *p* < 0.001 at the voxel level).

To assess whether dual-task-specific regions in the lPFC were associated with performance in the postural task, individual CoP displacements (relative task costs) were entered as covariate in the respective one-sample *t*-tests for single and dual one-back tasks. The respective correlation was visualized by averaging the beta values of all voxels obtained in this whole brain analysis and plotting these.

## Results

### Behavioral Data

#### Cognitive Performance

**FIGURE 2:**
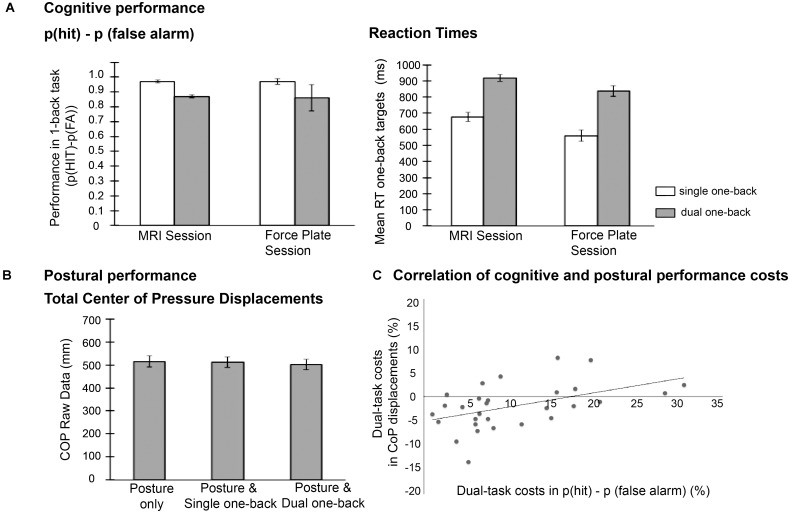
**(A)** Mean cognitive performance data, **(B)** mean postural performance (raw data), and **(C)** correlation of performance costs in both domains.

The repeated measures ANOVA on p(hit)-p(false alarm) performance with factors session (force plate vs. MRI) and task load (single vs. dual one-back task) revealed an effect of task load, *F* (1,20) = 70.0, *p* < 0.001, $\eta_{\text{p}}^{2}$ = 0.78, with lower performance in the dual one-back tasks [Mean (*M*) = 0.87, Standard error (SE) = 0.02] compared to the single one-back tasks (*M* = 0.97, *SE* = 0.02). In the whole group, the additional postural task did not affect this performance, as indicated by non-significant effects of session and interaction session × task load (see **Figure 2A**, left panel). Thus, while cognitive task load clearly deteriorated working-memory performance, the additional postural task did not in this sample of young participants.

The analysis of RT data also revealed an effect of task load, *F* (1,20) = 170.1, *p* < 0.001, $\eta_{\text{p}}^{2}$ = 0.90, with higher RTs in the dual one-back task (*M* = 877.73, *SE* = 33.32) compared to the single one-back task (*M* = 617.67, *SE* = 23.77; see **Figure 2A** right panel). Additionally, participants responded slower in the MRI session (*M* = 796.95, *SE* = 29.95) than in the force plate session [*M* = 698.45, *SE* = 25.71, *F*(1,20) = 59.28, *p* < 0.001, $\eta_{\text{p}}^{2}$ = 0.75]. Dual-task costs were more pronounced in the force plate session (mean difference single vs. dual task *M* = 278.10, *SE* = 21.18) than in the MRI session (*M* = 242.02, *SE* = 20.56) as indicated by the significant interaction effect of session × task load, *F*(1,20) = 8.57, *p* = 0.008, $\eta_{\text{p}}^{2}$ = 0.30.

#### Balance Performance

For the analysis of total CoP displacements (*n* = 29), relative performance costs were calculated in relation to the single postural task condition (dynamic fixation; see **Figure 2B** for absolute total COP displacement values). This analysis of performance costs revealed a significant difference between the postural condition with additional single one-back task performance (mean costs *M* = 2.47%, *SE* = 1.74) and the condition with additional dual one-back task (*M* = −1.72%, *SE* = 1.52, *t*(28) = 2.52, *p* = 0.018, *d* = 0.48). CoP values in both conditions did not differ significantly from the baseline condition (*p*’s > 0.16). Note, however, that the relative performance costs in the postural task varied strongly between individuals as indicated by the high standard errors.

#### Correlation Between Cognitive and Postural Performance Costs

To address the question whether dual-task costs in the cognitive domain (p(hit)–p(fa)) were associated with the triple-task costs in the postural domain, we correlated the cognitive dual-task costs from the MRI session with the COP costs in the force plate session. Both measures were correlated, *r* = 0.48, *p* = 0.008 (**Figure 2C**), suggesting that those individuals with high cognitive costs in the comparison of dual vs. single one-back tasks also had higher costs in the postural task when performed concurrently with the dual-one-back task on the force plate.

### Functional Imaging Data

#### Dual Task-Related Effects

**FIGURE 3:**
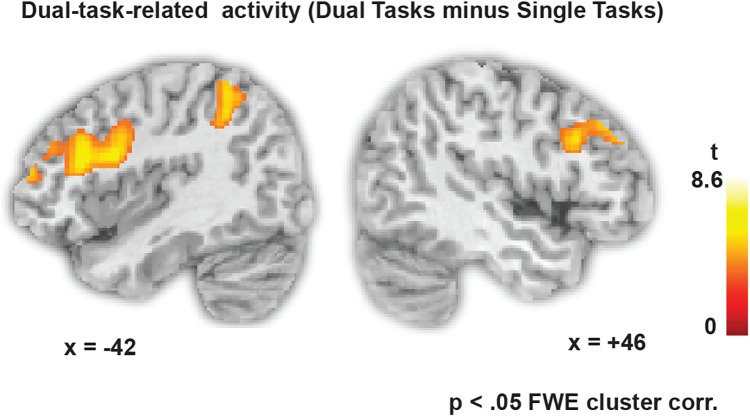
Dual-task-specific brain activity, revealed by contrasting all dual-task blocks with all single-task blocks (*p* < 0.05 FWE cluster-corrected, *p* < 0.001 at the voxel level).

**Figure 3** shows the dual-task-specific activity revealed by contrasting all dual-task blocks with all single-task blocks. The activity spans a fronto-parietal network involving bilateral lPFC as well as superior parietal regions. Also occipital and inferior temporal regions were more active in dual one-back working memory blocks than in single one-back blocks (see **Table 1** for all activity peaks).

**Table 1:** Activity peaks for the contrast dual tasks minus single tasks.

Region (labels for *k* > 10)	Hem	Brodman*n* Area	MNI coordinates			*T*-value	cluster size
			*x*	*y*	*z*		
Superior parietal lobule, precuneus, inferior parietal lobule, middle occipital gyrus, postcentral gyrus, superior occipital gyrus, supramarginal gyrus, angular gyrus	L	7, 40, 19, 5	−6 −30 −34	−70 −58 −48	58 50 40	8.64 8.60 7.10	3146 Included Included
Inferior temporal gyrus, middle temporal gyrus, middle occipital gyrus	L	37, 19	−52	−56	−8	7.75	389
Middle frontal gyrus, superior frontal gyrus, precentral gyrus	L	6	−30	2	68	7.62	454
Cerebellum	L/R		−4	−82	−26	7.04	846
			6	−84	−32	6.62	Included
Middle frontal gyrus, inferior frontal gyrus, precentral gyrus, superior frontal gyrus	L	9, 46, 10, 6	−40 −40 −38	28 10 52	20 24 12	6.09 5.98 5.55	1580 Included Included
Middle frontal gyrus, inferior frontal gyrus, superior frontal gyrus	R	9, 46, 10	46 50 44	20 28 40	30 34 32	5.30 5.24 4.86	520 Included Included
Superior parietal lobule, angular gyrus, inferior parietal lobule, precuneus, superior occipital gyrus, middle occipital gyrus	R	7, 40, 19	34 34 22	−60 −66 −74	50 42 52	5.16 4.85 3.61	416 Included Included

*p < 0.05 FWE cluster-corrected, p < 0.001 voxel level.*

#### Brain-Behavior Correlations

**FIGURE 4:**
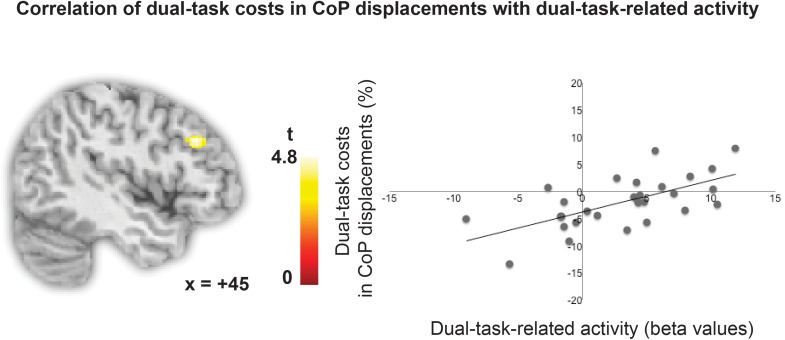
Correlation of relative costs in CoP displacements with dual-task-specific brain activity.

To test whether the degree of lPFC involvement in the high load dual one-back working memory task is related to the degree of performance costs in CoP displacements in the postural task, relative costs in total CoP displacements during dual one-back performance minus single one-back performance were entered as a covariate in the analysis. This analysis revealed that individual variability in the activity in a region in the right middle frontal gyrus in the mid-lPFC (*x* = 44, *y* = 30, *z* = 32, *k* = 59 voxels, *p* < 0.001, uncorrected) was positively correlated with the increase in relative costs in total CoP displacements while performing the dual one-back task on the force plate (see **Figure 4**). That is, individuals, who recruited the lPFC to a higher degree in a cognitive dual task, were less able to control their posture in addition as revealed by larger costs in CoP displacements. No such effect was present for the single one-back tasks. A *post hoc* analysis revealed that the right lPFC region overlapped partially with the dual-task-specific network identified in the group analysis (*k* = 35 voxels). In addition, the cluster partly overlapped (*k* = 36) with the *n*-back-associated right DLPFC region defined as a literature-based probabilistic region of interest by Heinzel et al. (2014). A small volume correction with this right DLPFC mask revealed that this sub-cluster was significant with *p* < 0.05 FWE-cluster corrected within this mask.

## Discussion

In the present fMRI study, we aimed to specify the contribution of the lPFC to interference processing in cognitive-motor multitasking situations. In a sample of healthy young adults, we showed a high degree of individual variability in (i) cognitive-motor interference between a postural task and a demanding dual one-back task on a behavioral level and in, (ii) lPFC recruitment during performance of the dual-one back task. Most importantly, we showed an association between these two variables – participants with higher interference costs in total CoP displacement were also characterized by higher dual-task-specific recruitment of the right middle frontal gyrus.

To our knowledge, this is the first study to show that a specific sub-region of the prefrontal cortex, i.e., the middle part of the right MFG is associated with cognitive-postural task interference in healthy young adults (but see Al-Yahya et al. (2016) for corresponding findings in a gait task in a stroke sample). This finding suggests an overlap between the resources required to successfully process interference between two cognitive tasks and between cognitive and postural tasks. It can be postulated that individuals with greater dual-task-specific lPFC recruitment have less neural capacity available to concurrently perform a postural task and vice versa. Note, that this association was not present for the single one-back task, which might be indicative of a load-dependent effect. Thus, even though lPFC activity during the postural task was not measured directly, the data from this association approach might support the notion that the lPFC is involved in postural control even in young adults.

Interestingly, lPFC activity was not directly related to cognitive task performance as measured by p(hit)-p(false alarm). This might indicate that some participants modulate their prefrontal activity in order keep an average performance level. This is in line with the neural efficiency hypothesis (Eysenck et al., 2007; Barulli and Stern, 2013), which denotes that less prefrontal neural recruitment during a comparable performance level may constitute a more efficient cognitive control system. This again enables participants to allocate more attention to concurrent tasks, resulting in better performance in this task. Also, during working memory tasks, an increase in fronto-parietal neural activation with increasing cognitive load has been described in terms of an adaptive mechanism in younger adults (Nagel et al., 2011; Heinzel et al., 2014; Heinzel et al., 2017). Accordingly, our findings further contribute to the idea that individuals who vary in their cognitive abilities might compensate for this by investing greater effort. This, in turn, might produce costs in additional tasks requiring overlapping resources, such as the postural task in the present study.

Another source of variability in cognitive-motor multitasking relates to prioritization strategies applied by individuals. As indicated in previous studies, complex multitasking environments may lead to a prioritization of one task over the other (Doumas et al., 2008). That is, the cognitive task receives priority over the postural task or vice versa. While older adults with cognitive impairments were found to prioritize the postural control task to avoid falling (i.e., posture first strategy; Rapp et al., 2006), this strategy of resource allocation does not seem to apply to younger adults as the risk of falling is almost negligible in this population (Granacher et al., 2011). In contrast, our results indicate that those young adults who operated at the limits of their resources rather focused on the performance of the cognitive task at the cost of impaired balance performance.

Additionally, the mean negative postural performance costs suggest that there was slightly greater postural sway in our sample in the posture only task, which was reduced when the demanding cognitive tasks were performed concurrently. There are various studies that report improved postural stability in postural-cognitive dual-task settings as compared to single postural tasks in young adults (Andersson et al., 2002; Riley et al., 2003). Task-specific changes in the direction of attention from an internal focus on the own body movement in the postural single task to the processing of visual and auditory stimuli in the cognitive-postural dual and triple tasks might provide one explanation for this effect. As suggested in the context of the ‘constrained action hypothesis’ (Wulf and Prinz, 2001; McNevin and Wulf, 2002; Wulf et al., 2004), focussing on the body movements might intervene with automatic postural control processes that maintain stable posture otherwise (Vuillerme and Nafati, 2007). This efficient automatic processing mode for the balance task might be re-established when attention is bound to the demanding cognitive tasks, thus leading to negative performance costs in the postural task. A combination of manipulations of attentional focus with neuroscientific measures might shed further light on these mechanisms in future studies.

Our fMRI results confirm previous findings of dual-task-specific neural activations in lateral fronto-parietal areas (D’Esposito et al., 1995; Schubert and Szameitat, 2003; Stelzel et al., 2006). Particularly activity in the lPFC has been related to dual-task-related increases in working memory load as well as processes associated with the coordination of the processing order of temporally overlapping tasks (Szameitat et al., 2002). The reported results of the present study do not allow to separate, whether individuals engage the right lPFC region more to deal with the higher working memory load or the flexible coordination demands for temporally overlapping tasks. Our previous study, which explicitly aimed at dissociating regions associated with load vs. coordination (Stelzel et al., 2008) suggests that regions in more anterior parts of the lPFC are rather related to dual-task coordination as compared to working-memory load associated with the number of relevant task rules. Also, further studies with other cognitive control paradigms suggest a role of the mid-lPFC in resolving interference in conflict situations (Botvinick et al., 2001; Miller and Cohen, 2001) but also related to high working memory load (Rypma et al., 1999; Curtis and D’Esposito, 2003; Nee et al., 2013). Future studies should specify this issue for cognitive-motor tasks, for example by varying the degree of temporal and structural overlap between cognitive and postural tasks.

A recent fMRI study directly assessed motor-cognitive dual-tasking in young and old adults using a simulated balance task concurrently with a calculation task (Papegaaij et al., 2017). Age-related differences in the up-regulation of activity from single to dual tasks were shown in the right insular cortex. However, no dual-task-specific activity was present for the applied dual task in that study. This suggests that task performance might not have involved higher working memory load or additional coordination processes, which in turn might be subject to inter-individual variability to a higher degree and thus might underly brain-behavior correlations. The cognitive dual task applied in our study revealed such dual-task-specific activity. Still, the association with postural sway remains an indirect one, as brain and behavioral measures were obtained in different sessions. This reflects the trade-off between using a naturalistic whole body balance task and obtaining anatomically precise online imaging data, which currently has to be resolved depending on the specific research question.

In contrast to studies with old adults, we did not find any robust interference effects in cognitive (i.e., in terms of p(hit)-p(false alarm)) or in balance performance on the group level. Only the RT data indicate that depending on the task load in the cognitive task (single vs. dual one-back task), interference with a postural task arises. When comparing RTs between the dual one-back task and the single one-back task dual-task costs were greater when the additional postural task was performed on the force plate than during MRI measurement. However, re-test effects cannot be excluded as an explanation for these differences in dual-task costs, as the MRI session took place after the force plate session for all participants. Previous studies on cognitive-motor interference mostly focused on old adults with fairly robust interference effects across studies (Woollacott and Shumway-Cook, 2002; Rapp et al., 2006; Granacher et al., 2011; Boisgontier et al., 2013). Findings in young adults are generally less consistent. While some studies showed cognitive-motor interference on a behavioral and a neural level in young adults (Holtzer et al., 2011; Zhou et al., 2014; Fujita et al., 2016), others failed to do so (Beurskens et al., 2014). Direct comparisons of the young and the aging brain have shown that old adults tend to show higher lPFC activity during working-memory tasks at lower objective loads compared to younger adults (Cappell et al., 2010). These findings suggest that due to degenerative processes, older adults might consistently engage additional resources (compensation-related utilization of neural circuits hypothesis, CRUNCH) to meet task demands (Reuter-Lorenz and Cappell, 2008). The resulting overactivation may have led to more stable results in the older population. The increased recruitment of right lPFC in young adults in the present study, suggests that also in the young population, some individuals might apply such compensatory processes to maintain an adequate performance level while others do not. The right lPFC thus might be a region that is recruited flexibly when individuals act at their capacity limits to support successful task performance under high load.

In sum, the present study allows preliminary insights into neural underpinnings of cognitive dual tasking in relation to balance performance in younger adults and suggests a possible mechanism, i.e., compensatory activity in right lPFC, that may explain a portion of variance in individual differences of balance performance. Characterizing the mechanisms of intra- and inter-individual differences in flexible resource allocation seems to be highly relevant for designing training procedures in impaired young and old adults. However, more research is needed to further understand personal as well as task factors that influence these individual differences.

## Limitations and Future Directions

Several limitations need to be considered when interpreting the results of this study. First, due to technical constraints, it was not possible to obtain data from the postural tasks during MRI testing. Even though we kept the tasks inside and outside the MRI as similar as possible, we were not able to show a direct relationship of the assumed compensatory recruitment of lPFC and CoP displacement. The shown association in the right lPFC might reflect the suggested common recruitment of this region for purely cognitive and cognitive-postural multitasking. Alternatively, as postural control has been associated with several other cortical and subcortical regions before, the shown association might be related to the extensive connectivity of the lPFC with other regions in terms of distant connectivity effects and thus be related to activity in other regions as well. Whether the right lPFC reflects dual-task specific processes (i.e., dual-task coordination or higher working memory load) or more general processes related to the allocation of limited resources cannot be inferred from our study and should be further addressed in the future.

Second, our sample was relatively small and replication in a larger sample would be important. With the advancement of neuroimaging techniques, a direct measurement of neural correlates of cognitive-motor multitasking interference may become feasible in future research.

Third, although we covered a range of input stimuli and output responses in the cognitive task, we do not have enough data to make conclusions about the generality of the shown association in the right lPFC. Also, differences between postural tasks and gait task should be further compared in future studies.

Regarding the implications for future training studies, our previous cognitive training study (Heinzel et al., 2016), indicated that over-activation in the right lPFC declined after 12 sessions of adaptive *n*-back working memory training. To present, however, it remains unclear if training-related alterations in lPFC may facilitate postural control performance likewise. Furthermore, it needs to be studied in future investigations, which specific training regimes lead to improvements in both cognitive performance and postural control. Possibly, an individualized motor-cognitive dual-task training that integrates multimodal diagnostic and evaluative parameters might be an effective approach.

## Conclusion

The current study investigated brain activation patterns during the performance of a cognitive dual task compared to a single task by using fMRI. In a second session outside the MRI scanner, the same task was applied using a postural control setting. Behavioral results of the cognitive dual task showed that RT but not performance level was affected by an additional postural task, indicating neural compensatory mechanisms. FMRI findings support this notion as increased lPFC activity was related to larger postural sway while cognitive task performance was kept at a comparable level. Findings of this study may improve our understanding of the underlying neural mechanisms during the performance of complex motor-cognitive multitask situations. Knowledge from this study could be used and implemented in training studies.

## Author Contributions

All authors listed have made a substantial, direct and intellectual contribution to the work, and approved it for publication.

## Conflict of Interest Statement

The authors declare that the research was conducted in the absence of any commercial or financial relationships that could be construed as a potential conflict of interest.

## Footnotes

1 https://www.neurobs.com/

2 http://www.fil.ion.ucl.ac.uk/spm

## References

[B1] Al-Yahya E. Johansen-Berg H. Kischka U. Zarei M. Cockburn J. Dawes H. (2016). Prefrontal cortex activation while walking under dual-task conditions in stroke: a multimodal imaging study. *Neurorehabil. Neural Repair* 30 591–599. 10.1177/1545968315613864 26493732 PMC5404717

[B2] Andersson G. Hagman J. Talianzadeh R. Svedberg A. Larsen H. C. (2002). Effect of cognitive load on postural control. *Brain Res. Bull.* 58 135–139. 10.1016/S0361-9230(02)00770-0 12121823

[B3] Barulli D. Stern Y. (2013). Efficiency, capacity, compensation, maintenance, plasticity: emerging concepts in cognitive reserve. *Trends Cogn. Sci.* 17 502–509. 10.1016/j.tics.2013.08.012 24018144 PMC3840716

[B4] Baudry S. (2016). Aging changes the contribution of spinal and corticospinal pathways to control balance. *Exerc. Sport Sci. Rev.* 44 104–109. 10.1249/JES.0000000000000080 27111478

[B5] Beurskens R. Helmich I. Rein R. Bock O. (2014). Age-related changes in prefrontal activity during walking in dual-task situations: a fNIRS study. *Int. J. Psychophysiol.* 92 122–128. 10.1016/j.ijpsycho.2014.03.005 24681355

[B6] Boisgontier M. P. Beets I. A. Duysens J. Nieuwboer A. Krampe R. T. Swinnen S. P. (2013). Age-related differences in attentional cost associated with postural dual tasks: Increased recruitment of generic cognitive resources in older adults. *Neurosci. Biobehav. Rev.* 37 1824–1837. 10.1016/j.neubiorev.2013.07.014 23911924

[B7] Botvinick M. M. Braver T. S. Barch D. M. Carter C. S. Cohen J. D. (2001). Conflict monitoring and cognitive control. *Psychol. Rev.* 108 624–652. 10.1037/0033-295X.108.3.624 11488380

[B8] Cappell K. A. Gmeindl L. Reuter-Lorenz P. A. (2010). Age differences in prefontal recruitment during verbal working memory maintenance depend on memory load. *Cortex* 46 462–473. 10.1016/j.cortex.2009.11.009 20097332 PMC2853232

[B9] Coren S. (1993). The lateral preference inventory for measurement of handedness, footedness, eyedness, and earedness: norms for young adults. *Bull. Psychon. Soc.* 31 1–3. 10.3758/BF03334122

[B10] Curtis C. E. D’Esposito M. (2003). Persistent activity in the prefrontal cortex during working memory. *Trends Cogn. Sci.* 7 415–423. 10.1016/S1364-6613(03)00197-9 12963473

[B11] D’Esposito M. Detre J. A. Alsop D. C. Shin R. K. Atlas S. Grossman M. (1995). The neural basis of the central executive system of working memory. *Nature* 378 279–281. 10.1038/378279a0 7477346

[B12] Doi T. Makizako H. Shimada H. Park H. Tsutsumimoto K. Uemura K. (2013). Brain activation during dual-task walking and executive function among older adults with mild cognitive impairment: a fNIRS study. *Aging Clin. Exp. Res.* 25 539–544. 10.1007/s40520-013-0119-5 23949972

[B13] Doumas M. Smolders C. Krampe R. T. (2008). Task prioritization in aging: effects of sensory information on concurrent posture and memory performance. *Exp. Brain Res.* 187 275–281. 10.1007/s00221-008-1302-3 18273609

[B14] Duncan J. Owen A. M. (2000). Common regions of the human frontal lobe recruited by diverse cognitive demands. *Trends Neurosci.* 23 475–483. 10.1016/S0166-2236(00)01633-7 11006464

[B15] Eysenck M. W. Derakshan N. Santos R. Calvo M. G. (2007). Anxiety and cognitive performance: attentional control theory. *Emotion* 7 336–353. 10.1037/1528-3542.7.2.336 17516812

[B16] Friston K. Holmes A. Worsley K. Poline J. Frith C. Frackowiak R. (1995). Statistical parametric maps in functional imaging: a general linear approach. *Hum. Brain Mapp.* 2 189–210. 10.1002/hbm.460020402

[B17] Fujita H. Kasubuchi K. Wakata S. Hiyamizu M. Morioka S. (2016). Role of the frontal cortex in standing postural sway tasks while dual-tasking: a functional near-infrared spectroscopy study examining working memory capacity. *Biomed Res. Int.* 2016:7053867. 10.1155/2016/7053867 27034947 PMC4791508

[B18] Fuster J. M. (2001). The prefrontal cortex–an update: time is of the essence. *Neuron* 30 319–333. 10.1016/S0896-6273(01)00285-9 11394996

[B19] Granacher U. Bridenbaugh S. A. Muehlbauer T. Wehrle A. Kressig R. W. (2011). Age-related effects on postural control under multi-task conditions. *Gerontology* 57 247–255. 10.1159/000322196 20980734

[B20] Heinzel S. Lorenz R. C. Brockhaus W. R. Wustenberg T. Kathmann N. Heinz A. (2014). Working memory load-dependent brain response predicts behavioral training gains in older adults. *J. Neurosci.* 34 1224–1233. 10.1523/JNEUROSCI.2463-13.2014 24453314 PMC6705311

[B21] Heinzel S. Lorenz R. C. Pelz P. Heinz A. Walter H. Kathmann N. (2016). Neural correlates of training and transfer effects in working memory in older adults. *Neuroimage* 134 236–249. 10.1016/j.neuroimage.2016.03.068 27046110

[B22] Heinzel S. Rimpel J. Stelzel C. Rapp M. A. (2017). Transfer effects to a multimodal dual-task after working memory training and associated neural correlates in older adults - a pilot study. *Front. Hum. Neurosci.* 11:85. 10.3389/fnhum.2017.00085 28286477 PMC5323430

[B23] Holtzer R. Mahoney J. R. Izzetoglu M. Izzetoglu K. Onaral B. Verghese J. (2011). fNIRS study of walking and walking while talking in young and old individuals. *J. Gerontol. A Biol. Sci. Med. Sci.* 66 879–887. 10.1093/gerona/glr068 21593013 PMC3148759

[B24] Jacobs J. V. Horak F. B. (2007). Cortical control of postural responses. *J. Neural Transm.* 114 1339–1348. 10.1007/s00702-007-0657-0 17393068 PMC4382099

[B25] Jahn K. Deutschlander A. Stephan T. Strupp M. Wiesmann M. Brandt T. (2004). Brain activation patterns during imagined stance and locomotion in functional magnetic resonance imaging. *Neuroimage* 22 1722–1731. 10.1016/j.neuroimage.2004.05.017 15275928

[B26] Jimura K. Locke H. S. Braver T. S. (2010). Prefrontal cortex mediation of cognitive enhancement in rewarding motivational contexts. *Proc. Natl. Acad. Sci. U.S.A.* 107 8871–8876. 10.1073/pnas.1002007107 20421489 PMC2889311

[B27] Kahneman D. (1973). *Attention and Effort.* Upper Saddle River, NJ: Prentice-Hall.

[B28] Lau T. M. Gwin J. T. Ferris D. P. (2014). Walking reduces sensorimotor network connectivity compared to standing. *J. Neuroeng. Rehabil.* 11:14. 10.1186/1743-0003-11-14 24524394 PMC3929753

[B29] Le Clair K. Riach C. (1996). Postural stability measures: what to measure and for how long. *Clin. Biomech.* 11 176–178. 10.1016/0268-0033(95)00027-5 11415618

[B30] Little C. E. Woollacott M. (2015). EEG measures reveal dual-task interference in postural performance in young adults. *Exp. Brain Res.* 233 27–37. 10.1007/s00221-014-4111-x 25273924 PMC4293276

[B31] Locke H. S. Braver T. S. (2008). Motivational influences on cognitive control: behavior, brain activation, and individual differences. *Cogn. Affect. Behav. Neurosci.* 8 99–112. 10.3758/CABN.8.1.99 18405050

[B32] McNevin N. H. Wulf G. (2002). Attentional focus on supra-postural tasks affects postural control. *Hum. Mov. Sci.* 21 187–202. 10.1016/S0167-9457(02)00095-7 12167298

[B33] Mihara M. Miyai I. Hatakenaka M. Kubota K. Sakoda S. (2008). Role of the prefrontal cortex in human balance control. *Neuroimage* 43 329–336. 10.1016/j.neuroimage.2008.07.029 18718542

[B34] Mihara M. Miyai I. Hattori N. Hatakenaka M. Yagura H. Kawano T. (2012). Cortical control of postural balance in patients with hemiplegic stroke. *Neuroreport* 23 314–319. 10.1097/WNR.0b013e328351757b 22357394

[B35] Miller E. K. Cohen J. D. (2001). An integrative theory of prefrontal cortex function. *Annu. Rev. Neurosci.* 24 167–202. 10.1146/annurev.neuro.24.1.167 11283309

[B36] Nagel I. E. Preuschhof C. Li S. C. Nyberg L. Backman L. Lindenberger U. (2011). Load modulation of BOLD response and connectivity predicts working memory performance in younger and older adults. *J. Cogn. Neurosci.* 23 2030–2045. 10.1162/jocn.2010.21560 20828302

[B37] Nee D. E. Brown J. W. Askren M. K. Berman M. G. Demiralp E. Krawitz A. (2013). A meta-analysis of executive components of working memory. *Cereb. Cortex* 23 264–282. 10.1093/cercor/bhs007 22314046 PMC3584956

[B38] Papegaaij S. Hortobagyi T. Godde B. Kaan W. A. Erhard P. Voelcker-Rehage C. (2017). Neural correlates of motor-cognitive dual-tasking in young and old adults. *PLoS One* 12:e0189025. 10.1371/journal.pone.0189025 29220349 PMC5722310

[B39] Papegaaij S. Taube W. Baudry S. Otten E. Hortobagyi T. (2014). Aging causes a reorganization of cortical and spinal control of posture. *Front. Aging Neurosci.* 6:28. 10.3389/fnagi.2014.00028 24624082 PMC3939445

[B40] Pashler H. (1994). Dual-task interference in simple tasks: data and theory. *Psychol. Bull.* 116 220–244. 10.1037/0033-2909.116.2.220 7972591

[B41] Peterka R. J. (2002). Sensorimotor integration in human postural control. *J. Neurophysiol.* 88 1097–1118. 10.1152/jn.00605.2001 12205132

[B42] Rapp M. A. Krampe R. T. Baltes P. B. (2006). Adaptive task prioritization in aging: Selective resource allocation to postural control is preserved in Alzheimer disease. *Am. J. Geriatr. Psychiatry* 14 52–61. 10.1097/01.Jgp.0000192490.43179.E7 16407582

[B43] Reisner V. Hinrichs D. (2016). *The Response Onset Tool (v1.0.0) [Software]. Zenodo.* Available at: https://zenodo.org/record/224317#.WySvk9IzbZ4

[B44] Reuter-Lorenz P. A. Cappell K. A. (2008). Neurocognitive aging and the compensation hypothesis. *Curr. Dir. Psychol. Sci.* 17 177–182. 10.1111/J.1467-8721.2008.00570.X

[B45] Riley M. A. Baker A. A. Schmit J. M. (2003). Inverse relation between postural variability and difficulty of a concurrent short-term memory task. *Brain Res. Bull.* 62 191–195. 10.1016/j.brainresbull.2003.09.012 14698352

[B46] Rypma B. Prabhakaran V. Desmond J. E. Glover G. H. Gabrieli J. D. (1999). Load-dependent roles of frontal brain regions in the maintenance of working memory. *Neuroimage* 9 216–226. 10.1006/nimg.1998.0404 9927550

[B47] Schubert T. Szameitat A. J. (2003). Functional neuroanatomy of interference in overlapping dual tasks: An fMRI study. *Cogn. Brain Res.* 17 733–746. 10.1016/S0926-6410(03)00198-8 14561459

[B48] Shumway-Cook A. Woollacott M. H. (2017). *Motor Control: Translating Research into Clinical Practice.* Philadelphia, PA: Lippincott Williams and Wilkins.

[B49] Stelzel C. Kraft A. Brandt S. A. Schubert T. (2008). Dissociable neural effects of task order control and task set maintenance during dual-task processing. *J. Cogn. Neurosci.* 20 613–628. 10.1162/jocn.2008.20053 18052784

[B50] Stelzel C. Schauenburg G. Rapp M. A. Heinzel S. Granacher U. (2017). Age-related interference between the selection of input-output modality mappings and postural control-a pilot study. *Front. Psychol.* 8:613. 10.3389/fpsyg.2017.00613 28484411 PMC5399084

[B51] Stelzel C. Schumacher E. H. Schubert T. D’Esposito M. (2006). The neural effect of stimulus-response modality compatibility on dual-task performance: an fMRI study. *Psychol. Res.* 70 514–525. 10.1007/s00426-005-0013-7 16175414

[B52] Szameitat A. J. Schubert T. Muller K. Von Cramon D. Y. (2002). Localization of executive functions in dual-task performance with fMRI. *J. Cogn. Neurosci.* 14 1184–1199. 10.1162/089892902760807195 12495525

[B53] Taube W. Gruber M. Gollhofer A. (2008). Spinal and supraspinal adaptations associated with balance training and their functional relevance. *Acta Physiol.* 193 101–116. 10.1111/j.1748-1716.2008.01850.x 18346210

[B54] Taubert M. Draganski B. Anwander A. Muller K. Horstmann A. Villringer A. (2010). Dynamic properties of human brain structure: learning-related changes in cortical areas and associated fiber connections. *J. Neurosci.* 30 11670–11677. 10.1523/JNEUROSCI.2567-10.2010 20810887 PMC6633410

[B55] Tombu M. Jolicoeur P. (2003). A central capacity sharing model of dual-task performance. *J. Exp. Psychol. Hum. Percept. Perform.* 29 3–18. 10.1037/0096-1523.29.1.3 12669744

[B56] Varghese J. P. Beyer K. B. Williams L. Miyasike-daSilva V. McIlroy W. E. (2015). Standing still: Is there a role for the cortex? *Neurosci. Lett.* 590 18–23. 10.1016/j.neulet.2015.01.055 25623039

[B57] Vuillerme N. Nafati G. (2007). How attentional focus on body sway affects postural control during quiet standing. *Psychol. Res.* 71 192–200. 10.1007/s00426-005-0018-2 16215747

[B58] Wickens C. D. (1980). “The structure of attentional resources,” in *Attention and Performance*, Nickerson R. (Hillsdale, NJ: Erlbaum).

[B59] Wittenberg E. Thompson J. Nam C. S. Franz J. R. (2017). Neuroimaging of human balance control: a systematic review. *Front. Hum. Neurosci.* 11:170. 10.3389/fnhum.2017.00170 28443007 PMC5385364

[B60] Woollacott M. Shumway-Cook A. (2002). Attention and the control of posture and gait: a review of an emerging area of research. *Gait Posture* 16 1–14. 10.1016/S0966-6362(01)00156-4 12127181

[B61] Wulf G. Mercer J. McNevin N. Guadagnoli M. A. (2004). Reciprocal influences of attentional focus on postural and suprapostural task performance. *J. Motor Behav.* 36 189–199. 10.3200/Jmbr.36.2.189-199 15130869

[B62] Wulf G. Prinz W. (2001). Directing attention to movement effects enhances learning: a review. *Psychon. Bull. Rev.* 8 648–660. 10.3758/Bf03196201 11848583

[B63] Zhou J. Hao Y. Wang Y. Jor’dan A. Pascual-Leone A. Zhang J. (2014). Transcranial direct current stimulation reduces the cost of performing a cognitive task on gait and postural control. *Eur. J. Neurosci.* 39 1343–1348. 10.1111/ejn.12492 24443958 PMC4221849

